# The TLR4-MyD88 Signaling Axis Regulates Lung Monocyte Differentiation Pathways in Response to *Streptococcus pneumoniae*

**DOI:** 10.3389/fimmu.2020.02120

**Published:** 2020-09-16

**Authors:** Rodrigo Sánchez-Tarjuelo, Isabel Cortegano, Juliana Manosalva, Mercedes Rodríguez, Carolina Ruíz, Mario Alía, María Carmen Prado, Eva M. Cano, María José Ferrándiz, Adela G. de la Campa, María Luisa Gaspar, Belén de Andrés

**Affiliations:** ^1^Immunobiology Department, Carlos III Health Institute, Madrid, Spain; ^2^Chronic Disease Programme, Carlos III Health Institute, Madrid, Spain; ^3^Bacterial Genetics Department, Carlos III Health Institute, Madrid, Spain; ^4^Consejo Superior de Investigaciones Científicas, Madrid, Spain

**Keywords:** TLR4, MyD88, *S. pneumoniae*, monocyte differentiation, innate immune responses, ROS

## Abstract

*Streptococcus pneumoniae* is the main cause of bacterial pneumonia, a condition that currently produces significant global morbidity and mortality. The initial immune response to this bacterium occurs when the innate system recognizes common motifs expressed by many pathogens, events driven by pattern recognition receptors like the Toll-like family receptors (TLRs). In this study, lung myeloid-cell populations responsible for the innate immune response (IIR) against *S. pneumoniae*, and their dependence on the TLR4-signaling axis, were analyzed in TLR4^–/–^ and Myeloid-Differentiation factor-88 deficient (MyD88^–/–^) mice. Neutrophils and monocyte-derived cells were recruited in infected mice 3-days post-infection. Compared to wild-type mice, there was an increased bacterial load in both these deficient mouse strains and an altered IIR, although TLR4^–/–^ mice were more susceptible to bacterial infection. These mice also developed fewer alveolar macrophages, weaker neutrophil infiltration, less Ly6C^high^ monocyte differentiation and a disrupted classical and non-classical monocyte profile. The pro-inflammatory cytokine profile (CXCL1, TNF-α, IL-6, and IL-1β) was also severely affected by the lack of TLR4 and no induction of Th1 was observed in these mice. The respiratory burst (ROS production) after infection was profoundly dampened in TLR4^–/–^ and MyD88^–/–^ mice. These data demonstrate the complex dynamics of myeloid populations and a key role of the TLR4-signaling axis in the IIR to *S. pneumoniae*, which involves both the MyD88 and TRIF (Toll/IL-1R domain-containing adaptor-inducing IFN-β) dependent pathways.

## Introduction

*Streptococcus pneumoniae* (pneumococcus) is a Gram-positive bacterium that colonizes and invades the respiratory tract. It is the main etiological agent of community acquired pneumonia, accounting for about 90% of all pneumonia deaths, especially in young children and the elderly (1, 2). As a consequence, it is a major cause of morbidity and mortality worldwide (2), although the present pandemic induced by SARS-Cov2 is currently changing the top list of the most dangerous respiratory pathogens (3). Globally, *S. pneumoniae* causes over 800,000 deaths in children and 13.8 million cases of pneumonia each year. Different vaccines have been generated against the most prevalent *S. pneumoniae* serotypes, of which the conjugate 13-valent (PCV13) and the polysaccharide-based 23-valent (PCV23) are often used (4). The use of antibiotics is becoming compromised by the ever-increasing appearance of antibiotic-resistant strains (5–7). Moreover, as the immune lung response underlying *S. pneumoniae* infection is still not fully understood, a better understanding of this response will be essential to design new effective therapeutic interventions.

It is known that *S. pneumoniae* recognition by lung epithelial cells and by the innate immune system (IIS) involves several pattern recognition receptors (PRRs), receptors that are expressed in different cell lineages and that recognize pathogen associated molecular patterns (PAMPs). Toll-like receptors (TLRs) constitute one of the most important PRR families, playing a critical role in initiating inflammatory responses and promoting adaptive immune responses (8, 9). Of these, both TLR2 and TLR4 may participate in the innate immune response (IIR) against *S. pneumoniae*. TLR2 recognizes lipoteichoic acid (LTA), peptidoglycans and lipopeptides, components of the Gram-positive cell wall (10, 11), although as it appears to play a limited role in the IIR against *S. pneumoniae* other TLRs are likely to be implicated in this process (12). TLR4 plays an essential role in the host’s defense against Gram-negative bacteria by recognizing lipopolysaccharide (LPS) (13), although this is not presented by *S. pneumoniae*. Nevertheless, it also recognizes LTA and pneumolysin (Ply), the latter a pneumococcal enzyme that is released by these bacteria. Hence, TLR4 may participate in the response against *S. pneumoniae* (13, 14), and the interaction between Ply and TLR4 during pneumococcal colonization of the nasopharynx may be important for protection (14). After ligand binding, TLR2 and TLR4 depend on the signaling adaptor protein MyD88 (Myeloid-Differentiation factor-88) for activation. In addition, TLR4 can signal through both MyD88- and TRIF-dependent (Toll/IL-1R domain-containing adaptor-inducing IFN-β) pathways (8, 9). Moreover, MyD88^–/–^ mice infected with *S. pneumoniae* generate a stronger bacterial burden and a weaker inflammatory response in the lung (15).

Alveolar macrophages (AMs) are tissue-resident macrophages (Mϕ) that colonize lung tissues during embryogenesis and that are thereafter maintained through self-renewal (16). Alveolar macrophages initiate lung IRs by recognizing and phagocytosing *S. pneumoniae*, and they are essential to control bacterial numbers in the first hours after infection. However, due to the limited number of AMs in the alveoli, the efficacy of AM-mediated immunity against *S. pneumoniae* depends on the magnitude of the bacterial inoculum (16, 17). After antigen-activation, AMs release different cytokines (TNF-α, IFN-γ, GM-CSF, and IL-6) that attract neutrophils (Nϕ) and monocytes, promoting their infiltration into the lung parenchyma (17). Natural killer (NK) cells also play an important role in the immune response against *S. pneumoniae*, producing IFNγ in the early stages of lung infection, and further favoring the activation and recruitment of Nϕ to the lung (18). Nϕ are the most abundant myeloid cell population in the lung and their lung infiltration is fundamental for bacterial clearance, particularly since it is responsible for bacterial phagocytosis and complement activation (19). Furthermore, bone marrow-derived populations of Ly6C^hi^ monocytes migrate to peripheral tissues like the lung or the spleen in response to inflammatory stimuli or to the lesions caused by *S. pneumoniae*, and in a CCR2-dependent manner (20). Once recruited into peripheral tissues, Ly6C^hi^ monocytes can further differentiate into monocyte-derived dendritic cells (moDCs) and monocyte-derived Mϕ (moMϕ), two populations that participate in bacterial removal in conjunction with Nϕ (20, 21).

Mononuclear phagocytic cells are quite heterogeneous and they display significant plasticity, which allow them to acquire specialized functions. In blood, two types of monocytes have been identified with differential phenotype and functions. The classical monocytes (cMO) are CD11b^+^Ly6C^hi^CX3CR1^lo^CD43^lo^, are found in tissues under homeostasis, and during inflammation they are recruited to the damaged tissues and produce proinflammatory cytokines such as TNF-α and IL6, as well as enhancing the inducible nitric oxide synthase (iNOS) available. These classical inflammatory monocytes participate in the local innate immune control of several pathogens, such as *Toxoplasma gondii, Leishmania donivani, Leishmania major* and *Listeria monocytogenes* (22–25). By contrast, the non-classical monocytes (ncMO) monocytes are CD11b^+^Ly6C^lo^CX3CR1^hi^CD43^hi^, they patrol the endothelium and have been shown to have angiogenic activity by secreting vascular endothelial growth factor (VEGF) and IL-10. These ncMO monocytes are also recruited to the damaged tissues and may be involved in tissue repair and wound healing (20, 26–28). In the inflammed tissues, recruited monocytes differentiate into Mϕ. Two phenotypes of Mϕ have been proposed that are related to their functional profile (29–31). Upon activation in response to proinflammatory cytokines like TNF-α and IFN-γ, monocytes differentiate to Mϕ that adopt the M1 phenotype with enhanced microbicidal activity, and secrete pro-inflammatory cytokines (30). By contrast, M2 Mϕ are induced by the Th2-related cytokines (IL-13 and IL-4) and they secrete anti-inflammatory factors like IL-10 and TGF-β. Thus, M2 Mϕ participate in tissue repair and in the phagocytosis of apoptotic cells, limiting inflammation (32). The equilibrium between the M1 and M2 populations is essential to produce an appropriate IR, including its resolution. *S. pneumoniae* infection induces an inflammatory response mediated by M1 Mϕ, killing pathogens and triggering adaptive immunity. Autolysis of *S. pneumoniae* leads to the production of Ply and it induces the production of reactive oxygen species (ROS) by Nϕ (33). Likewise, other bacterial infections like those caused by *Mycobacterium tuberculosis*, induce TLR-activation of Mϕ, bacterial phagocytosis and intense ROS production as the main core of the IIR against such infection (34, 35).

Here we examined the dynamics of the lung IIR against *S. pneumoniae*, using a clinical isolate obtained from peripheral blood and analyzing the myeloid populations that accumulate, their markers of activation and their dependency on the TLR4-MyD88 pathway after intranasal administration. Our data show that 3 days post infection (dpi) there is a local increase in the Nϕ, monocytes, and the moMϕ and moDC monocyte-derived populations, with a prominent cMO profile. In the absence of TLR4 or MyD88, the production of these myeloid populations was dampened. The ROS produced by different myeloid cell types highlighted the importance of the TLR4-MyD88 axis in the oxidative burst within the myeloid compartment. Moreover, the lung cytokine profile and the production of ROS were less affected by infection in MyD88^–/–^ mice than in TLR4^–/–^ mice, although these responses were weaker than in infected WT mice. Hence, an alternative TRIF pathway appears to participate in the IIR against *S. pneumoniae*. Together, these data indicate that both TLR4-MyD88 and TLR4-TRIF signaling are involved in the recognition of *S. pneumoniae* by the cells generated through the myeloid IIR in the lung, highlighting the role of these pathways in the cytotoxic and regulatory responses that combat this bacterium.

## Materials and Methods

### Mice

Adult (8–10 weeks old) WT (C57BL/6 and C57BL/10), C57BL/10/TLR4^–/–^ and C57BL/6/MyD88^–/–^ mice were bred and maintained at the animal facility of the Centro Nacional de Microbiología-Instituto de Salud Carlos III (CNM-ISCIII, Madrid, Spain). All animal experiments were approved by the Institutional Review Board at the ISCIII, and carried out in strict accordance with EU and National Animal Care guidelines (directive 2010/63/EU and RD 53/2013). The experimental protocol was also approved by the Consejería de Medio Ambiente Comunidad de Madrid (PROEX110/15, PROEX021/18).

### Induction of Infection

**FIGURE 1:**
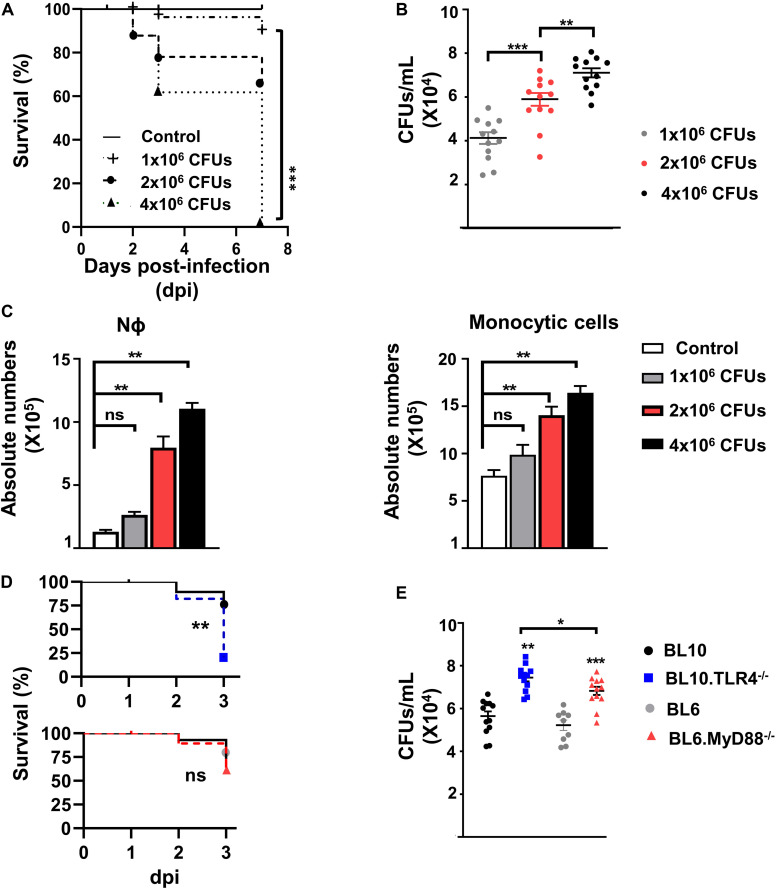
Dose-response study following *S. pneumoniae* bacterial infection of the lungs. **(A)** Survival of WT.BL10 mice after intranasal infection with different concentrations of *S. pneumoniae* CFUs. Survival was determined at 2, 3, and 7 dpi, and the differences between the groups were compared using Kaplan–Meier tests. The data represent measurements from three independent replicates: Control (*n* = 6); 1 × 10^6^ CFUs (*n* = 8); 2 × 10^6^ CFUs (*n* = 14); 4 × 10^6^ CFUs (*n* = 10). Statistical analysis was performed using a log-rank Mantel–Cox test: ***p < 0.001. **(B)** CFUs determined in lung suspensions from infected mice at 72 h pi. Suspensions were plated on sheep blood agar plates and incubated for 24 h at 37°C in 5% CO_2_. The data represent the individual measurements of three independent experiments. Shown inside are means ± SEM; *n* = 12. **(C)** Quantification of Nϕ (Gr1^hi^CD11b^hi^) and monocyte cell populations (Gr1^–^CD11b^lo/hi^) in whole lung cell suspensions (without ACK treatment) obtained at 3 dpi following infection with 1 × 10^6^, 2 × 10^6^ and 4 × 10^6^ CFUs (as defined in Supplementary Figure 1). Bar graphs show the absolute numbers of each population calculated from the frequencies obtained by flow cytometry and the total number of cells counted in each sample in three independent experiments. Data are means ± SEM; control (*n* = 6); 1 × 10^6^ CFUs (*n* = 8); 2 × 10^6^ CFUs (*n* = 14); 4 × 10^6^ CFUs (*n* = 10). **(D)** Survival of WT.BL10 (*n* = 12), TLR4^–/–^ (*n* = 12), WT.BL6 (*n* = 10) and MyD88^–/–^ (*n* = 12) mice after intranasal infection with 2 × 10^6^ CFUs. Survival was assessed at 1, 2, and 3 dpi and the differences between the groups were compared using a log-rank Mantel–Cox test. **(E)** Colonies formed from the lung suspensions were counted at 3 dpi as in panel B; WT.BL10 (n = 12), TLR4^–/–^ (*n* = 12), WT.BL6 (*n* = 9) and MyD88^–/–^ (*n* = 12). The data in panels **(B,C,E)** were compared among multiple groups with one-way ANOVA and unpaired two tailed Student’s *t*-test. *p < 0.05, **p < 0.01, ***p < 0.001, ns, not significant.

Mice were lightly anesthetized with an aerosol containing 4% isoflurane (Zoetis, United Kingdom) and they were inoculated intranasally with a suspension (20 μL in Phosphate Buffered Saline –PBS: Bio-Whittaker, Lonza) of the *S. pneumoniae* bacterial strain 1195 (serotype 3 and ST260 genotype). Strain 1195 is a clinical isolate obtained from peripheral blood that was obtained from a septic patient and submitted to the Spanish Pneumococcus Reference Laboratory. This 1195 strain was described previously (referred to as CipR1) (36) and other strains of the ST260 genotype have been used previously in a rabbit model of experimental meningitis (37). The pneumococcal strain was grown to mid-log growth phase in Todd-Hewitt broth supplemented with 0.5% yeast extract. The bacteria were then recovered by centrifugation and suspended in medium with 25% glycerol at a concentration of 10^8^ cells/mL, and stored as aliquots at −80°C. For intranasal infection, aliquots were thawed, washed and resuspended in PBS immediately before use. We performed a dose-response study with this strain from 1 to 4 × 10^6^ colony-forming units (CFUs) to determine the optimal concentration required to mount an immune response (Figure 1A).

### Determination of the Colony-Forming Units

The lungs of the mice were dissected out at 1, 2, and 3 dpi, and cell suspensions were prepared by mechanical dissociation in 3 mL of cold (4°C) staining buffer (2.5% Fetal Calf’s Serum in PBS). No enzymatic digestion by collagenase treatment was used, since preliminary experiments showed an important reduction in the recovery of myeloid-gated cells and CD11b+ cells after such treatment (Supplementary Material). The lung tissue was cut into small pieces and cell suspensions were prepared by mechanical dissociation, disrupting the tissue and filtering through a 40 μm pore cell strainer (BD Biosciences). The filtrate was then centrifuged for 5 min at 110 *g* and 4°C in order to obtain the lung cells. The material recovered was analyzed by flow cytometry (see below), and the *S. pneumoniae* CFUs and cytokines in the supernatants were quantified. Colony-forming units were determined by growing serial dilutions of the lung supernatant on Mueller-Hinton sheep blood agar plates (BD Biosciences, San Jose, CA), counting the colonies formed after a 24 h incubation at 37°C in an atmosphere of 5% CO_2_. Dissemination of the bacteria to other organs was determined by CFU quantification in samples from spleen and olfactory bulbs at 3 dpi (50 ± 7 CFUs, *n* = 6 in splenic samples of infected BL10).

### Flow Cytometry

Lung cell suspensions obtained from the entire lung were evaluated by flow cytometry, employing a multi-panel strategy to distinguish the different myeloid cell populations in the samples. Cell pellets from lung homogenates were treated for 2 min at room temperature (RT) with 1 mL ACK buffer (Potassium Bicarbonate: Life Technologies) to lyse the erythrocytes. The cells (4 × 10^6^/200 μL) were then washed twice with staining buffer, recovered by a 5 min centrifugation at 110 *g* and 4°C, and then prepared in staining buffer. Cell viability was measured by Trypan blue (Merck) dye exclusion and viable cells were counted in a Neubauer’s chamber. Non-specific antibody binding was blocked by incubation for 10 min at 4°C with an anti-Fc-Block (clone 2.4G2: BD Biosciences) and the cell populations were then analyzed by Flow Cytometry after staining for 20 min at 4°C using the specific mAbs listed in Supplementary Table 1. Dead cells were discharged by staining with Fixable LIVE/DEAD violet-510 kit (Thermo Fisher Scientific) and the cells were fixed for 15 min at RT in 2% paraformaldehyde before acquisition on a LRS Fortessa X-20 cytometer (BD Biosciences). The samples were analyzed using the DIVA v8.0 software package (BD Biosciences) and the cell populations were analyzed after electronic gating on the basis of SSC-A and FSC-A, followed by a gating strategy to rule out doublets and dead cells (Supplementary Figure 1). At least 1–3 × 10^5^ live cells were analyzed in each sample and the mAbs used in this study allowed the identification of lung myeloid populations, distinguishing the AM (Siglec-F^hi^CD11b^lo^), NK (CD3^–^NK1.1^+^), Nϕ (Gr1^hi^CD11b^hi^), monocytes (Gr1^–^ CD11b^hi^F4/80^+^CD11c^lo^), moMϕ (Gr1^–^CD11b^hi^F4/80^lo^CD11c^+^Ly6C^–^), and moDC (CD11b^hi^F4/80^lo^CD11c^+^Ly6C^+^) populations. Finally, the differential expression of Ly6C and Ly6G was used to characterize the CD11b^+^ monocytic populations (22, 25, 27, 28): cMO (CD11b^hi^Ly6C^hi^Ly6G^–^) and ncMO (CD11b^hi^Ly6C^lo^Ly6G^–^). Isotype fluorescence minus one (FMO) controls were performed to rule out non-specific fluorescence and to define gating boundaries (Supplementary Figure 1).

### Cytometric Bead Array for Cytokines

Supernatants obtained from fresh lung suspensions (total volume 3 mL) were aliquoted and frozen at −80°C. The aliquots were thawed (only once) and the cytokines present in a 25 μL sample were analyzed (CXCL1, IL-18, IL-23, IL-12p70, IL-6, TNF-α, IL-1β, and IL-12p40) with the LEGENDplex Cytokine Analysis kit (BioLegend), according to the manufacturer’s recommendations, and using a FACS Canto I cytometer (BD Biosciences) and the BioLegend LEGENDplex software.

### Quantitative Real-Time PCR

Total RNA from lung samples was extracted using NucleoZol (Macherey-Nagel) and reverse transcribed in a final volume of 25 μL with Oligo(dT)-primers, as described previously (38). The cDNA (1 μL) obtained was amplified quantitatively by real-time PCR (qPCR) on a CFX96^TM^ Real-Time System using the SsoFast^TM^SupermixEvaGreen (Bio-Rad, Hercules, CA), as indicated elsewhere (39). The Bio-Rad CFX Manager software was used to calculate the C_T_ of each reaction and the specific amount of cDNA in each sample was determined relative to the expression of the *hypoxanthine phosphoribosyl transferase-1* (HPRT) gene by the 2^–ΔCt^ method (40). The primers used in this study are listed in Supplementary Table 2.

### Detection of Intracellular Reactive Oxygen Species

The ROS-sensitive probe 2′,7′-dichlorodihydrofluorescein diacetate (H_2_DCFDA, 10 mM stock solution: Invitrogen) was used here on fresh cells surface-labeled as described in the Flow Cytometry section but without fixation procedure. The cells were then labeled for 40 min in the dark at RT with H_2_DCFDA (5 μM in PBS), washed twice with PBS and recovering the cells by centrifugation for 5 min at 4°C and 110 *g*, and resuspended them in a final volume of 1 mL. The labeled cells were incubated with 2 × 10^6^ CFUs of heat-inactivated *S. pneumoniae* (60 min, 60°C) for 15, 30, and 60 min at 37°C, and they were then analyzed by flow cytometry to measure the ROS signals of the different myeloid populations. The baseline fluorescence of the cells was determined prior to their exposure to *S. pneumoniae*.

### Statistical Analysis

The data are presented as the means ± SEM (Standard error of the mean). Statistical analyses were performed with the Prism 8.0 (Graph Pad) software, after testing the normality of the data distributions with the Kolmogorov–Smirnov and D’Agostino-Pearson tests. The data were compared with one-way ANOVA tests and two-tailed unpaired Student’s *t*-tests. Survival curves were obtained using Kaplan–Meier tests and analyzed using a log-rank (Mantel–Cox) test. A value of *p* < 0.05 was considered statistically significant: *p < 0.05; **p < 0.01; ***p < 0.001; ****p < 0.0001; ns, not significant.

## Results

### Innate Immune Responses of Lung Cell After *S. pneumoniae* Infection

We performed initial experiments to establish the optimal dose for *S. pneumoniae* serotype 3 strain 1195 infection and the time required to mount an IIR, parameters that allowed us to study the myeloid cell subpopulations involved, as well as the role of TLR4 and MyD88 expression. Adult WT.BL10 mice were infected intranasally with increasing doses of bacteria (1 × 10^6^, 2 × 10^6^, and 4 × 10^6^ CFUs) and their survival after infection was studied. A dose-dependent decrease in survival was found from 3 dpi, accompanied by an increase in bacterial CFUs in the lungs (Figures 1A,B). It is known that Nϕ and monocyte cell infiltration is essential for *S. pneumoniae* lung clearance (12, 41). Enhanced recruitment of Nϕ (Gr1^hi^CD11b^hi^) and monocytes (Gr1^–^CD11b^lo/hi^) to the lung was evident at 3 dpi in mice that received 2 × 10^6^ and 4 × 10^6^ CFUs (Figure 1C). Thus, we chose the dose of 2 × 10^6^ CFUs to infect and monitor the lung IIR at 3 dpi. Previous studies have shown that pneumococcal LTA and Ply were recognized by TLR2 and TLR4, as the main PRRs required to initiate the immune response (13, 14) and acting through the MyD88-signaling cascade. To analyze the contribution of TLR4 and MyD88 signaling to the IIR, TLR4^–/–^ and MyD88^–/–^ mice were infected intranasally with 2 × 10^6^ CFUs *S. pneumoniae* (Figures 1D,E), and their survival and bacterial load was quantified at 1, 2, and 3 dpi (Figure 1E and Supplementary Figure 2). Since the different genetic backgrounds of the TLR4^–/–^ (BL10) and MyD88^–/–^(BL6) mice may influence their IIR against *S. pneumoniae*, we used both WT.BL10 and WT.BL6 control strains, with no differences in survival and bacterial load detected between them. The TLR4^–/–^and MyD88^–/–^ mice survived less time after infection than their corresponding WT animals (WT.BL10 and WT.BL6, respectively), although the TLR4^–/–^ mice survived worse than the MyD88^–/–^ mice at this CFU dose. Accordingly, the bacterial load was higher in the lungs of infected TLR4^–/–^ than in the MyD88^–/–^ mice, which both exceeded that in the WT infected mice (Figure 1E and Supplementary Figure 2).

**FIGURE 2:**
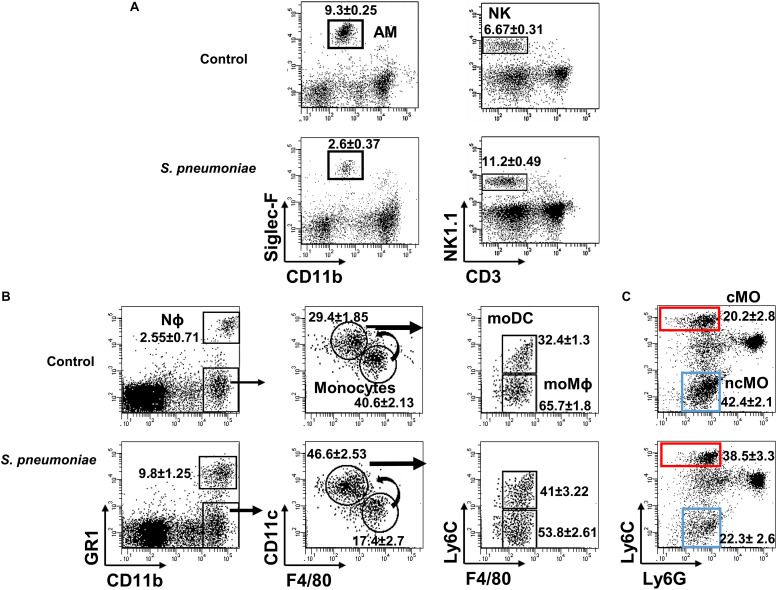
Lung myeloid populations after *S. pneumoniae* infection. Lung suspensions from infected and uninfected WT.BL10 mice were prepared at 1 and 3 dpi, and stained with the indicated mAbs (see section “Materials and Methods”) **(A)** Samples obtained at 1 dpi were stained with anti-CD11b and Siglec-F to identify AM (Siglec-F^hi^CD11b^lo^) cells in the left dot plots. NK cells were identified using anti-NK1.1 and anti-CD3 antibodies: NK (NK1.1^+^CD3^–^) in the right dot plots. **(B)** At 3 dpi, myeloid and granulocyte cell populations were discriminated using the markers CD11b and Gr1, as described in Supplementary Figure 1 in which the sequential gating strategy to identify distinct monocyte populations is depicted. **(C)** The cMO and ncMO profile of monocytes was defined based on their Ly6C and Ly6G expression: cMO (CD11b^hi^Ly6C^hi^Ly6G^–^, red box) and ncMO (CD11b^hi^Ly6C^lo^Ly6G^–^, blue box). Representative dot plots are shown and the numbers inside are the percentages mean ± SEM of four independent experiments, WT.BL10 (*n* = 12).

**FIGURE 3:**
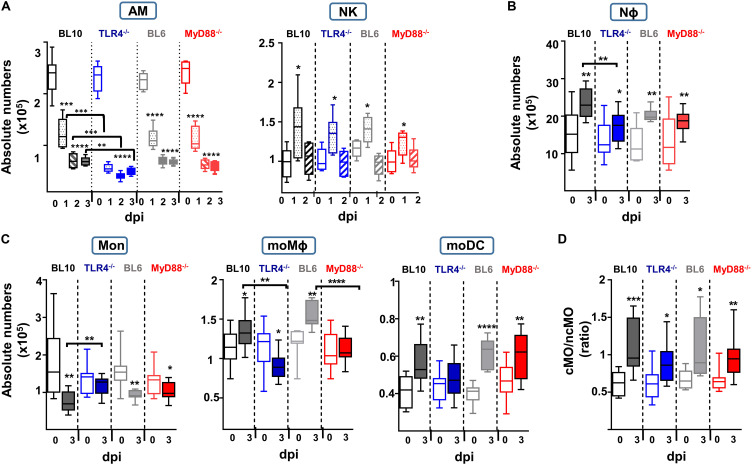
Quantification of the absolute numbers of different lung innate subsets after *S. pneumoniae* (2 × 10^6^ CFUs) instillation to WT.BL10, TLR4^–/–^, WT.BL6 and MyD88^–/–^ mice. **(A)** AM and NK cells were identified and counted at 0, 1, 2, and 3 dpi. Absolute numbers were calculated using the frequencies obtained by cytometry and the total number of cells counted in each sample: WT.BL10 (*n* = 6), TLR4^–/–^ (*n* = 6), WT.BL6 (*n* = 6), and MyD88^–/–^ (*n* = 6) at 0, 1, 2, and 3 dpi. **(B–D)** At 3 dpi the IIR was analyzed by quantifying the Nϕ cells, monocytes, the moDC and moMϕ cells, and the cMO and ncMO populations from control and infected mice. Box-and-whisker plots show the median values, with the bottom and top of the box indicating the first quartile to the third quartile, with the minimum and maximum values from four independent experiments: WT.BL10 (*n* = 12), TLR4^–/–^ (*n* = 15), WT.BL6 (*n* = 6), and MyD88^–/–^ (*n* = 15). All the populations were analyzed by one-way ANOVA and with an unpaired two-tailed Student’s *t*-test: *p < 0.05, ** *p* < 0.01, *** *p* < 0.001, **** *p* < 0.0001.

Resident AMs (Siglec-F^hi^CD11b^lo^) are essential to control bacterial numbers early after *S. pneumoniae* infection, driving a reduction in AM numbers by inducing their caspase-dependent apoptosis (16, 17, 42, 43). Natural killer (CD3^–^NK1.1^+^) cells also exert their effector and regulatory functions soon after infection. Accordingly, we found fewer AMs in the lungs of both infected WT strains on days 1–3 pi, along with a transient accumulation of NK cells in the lung at 1 dpi (Figures 2A, 3A). Myeloid-cells in the lung were analyzed by flow cytometry at 3 dpi (Figures 2B, 3B) and as expected, there was significant Nϕ recruitment in the infected lungs, coupled to a decrease in monocytes. Indeed, a decrease in F4/80 (a specific marker of mature Mϕs) and an increase in CD11c was observed, indicating the activation and differentiation of these mature Mϕs. When Ly6C expression was analyzed in the differentiated CD11c^hi^F4/80^lo^ population, an increase in the absolute numbers of both moMϕ and moDC was seen in infected mice (Figure 2B right dot plots, Figure 3C). The cMO/ncMO profile was defined on gated CD11b^+^ cells, detecting differential Ly6C and Ly6G expression as described previously. Monocytes in control and infected mice shifted toward a pro-inflammatory cMO/ncMO ratio (BL10 1.12 ± 0.09, *n* = 12; BL6 1.073 ± 0.174, *n* = 6) in the infected lungs when compared to the control non-infected mice (BL10 0.61 ± 0.04, *n* = 12; *p* < 0.001; BL6 0.68 ± 0.05, *n* = 6 *p* < 0.05; Figures 2C, 3D).

Together these data reveal distinct local cell dynamics in the lungs of *S. pneumoniae* infected mice, and both WT.BL10 and WT.BL6 control strains behave similar in terms of myeloid cell recruitment. These changes involved an initial decrease in AM cells during the first 3 dpi, along with the accumulation of NK cells on day 1. Subsequently, there was neutrophil infiltration and monocyte differentiation toward the moMϕ and moDC phenotypes at 3 dpi, with a predominant cMO profile.

### Defective Innate Response to *S. pneumoniae* in Infected TLR4 and MyD88 Deficient Mice

The survival of TLR4^–/–^ and MyD88^–/–^ infected mice was worse than that of WT mice, and they had a higher bacterial load, especially the TLR4^–/–^ mice (Figures 1D,E). As in WT.BL10 and WT.BL6 mice, *S. pneumoniae* infection produced an accumulation of NK cells and an important reduction in AM cells in TLR4^–/–^ and MyD88^–/–^ mice (Figure 3A), with the decrease in AM cells more pronounced in TLR4^–/–^ infected mice than in either of the WT strains or the MyD88^–/–^ mice (TLR4^–/–^ versus MyD88^–/–^ values *p* < 0.001 and *p* < 0.01 at days 1 and 2 pi, respectively). There was similar recruitment of NK cells to the lungs at 1 dpi in the four strains of mice. By contrast, deficient accumulation of Nϕ was detected by 3 dpi in TLR4^–/–^ mice relative to the WT.BL10 infected mice (Figure 3B).

Although the lungs from infected WT.BL10 and WT.BL6 mice have fewer monocytes due to their differentiation toward a moMϕ and moDC populations (Figure 2B, right dot plots), there was only a minimal reduction in the monocyte populations in infected TLR4^–/–^ mice (Figure 3C), with no increase in their moDC population and a decrease in moMϕ number. By contrast, there was a reduction in monocytes in MyD88^–/–^ infected mice and differentiation toward the moDC lineage was observed, as occurred in WT.BL6 animals, although there was no increase in the moMϕ cell population that remained much lower than in the WT.BL6 mice (Figure 3C). Upon infection, and based on the Ly6C expression (20, 22, 26, 28) the cMO/ncMO profile shifted slightly toward a pro-inflammatory profile in TLR4^–/–^ mice (0.64 ± 0.05 uninfected, 0.89 ± 0.079 infected mice, *n* = 15; *p* < 0.05), whereas this ratio was higher in MyD88^–/–^ mice (0.68 ± 0.04 uninfected mice, 1.003 ± 0.087 infected, *n* = 15; *p* < 0.01), similar to that found in WT.BL10 and WT.BL6 animals. In parallel, there was an important reduction in adaptive B cell and CD4+ T cell recruitment to the lungs of TLR4^–/–^ and MyD88^–/–^ infected mice at 3 dpi, whereas CD8^+^ cells were recruited normally (manuscript in preparation).

In summary, these findings demonstrate important defects in the local lung IIR in MyD88^–/–^ and TLR4^–/–^ mice, with a more severe phenotype in the latter model. These changes included important defects in AM, NK, and Nϕ cell recruitment, as well as significant defects in monocyte differentiation and the adaptive response.

### Differential Impairment of Inflammatory Cytokine Production in TLR4^–/–^ and MyD88^–/–^ Infected Mice

**FIGURE 4:**
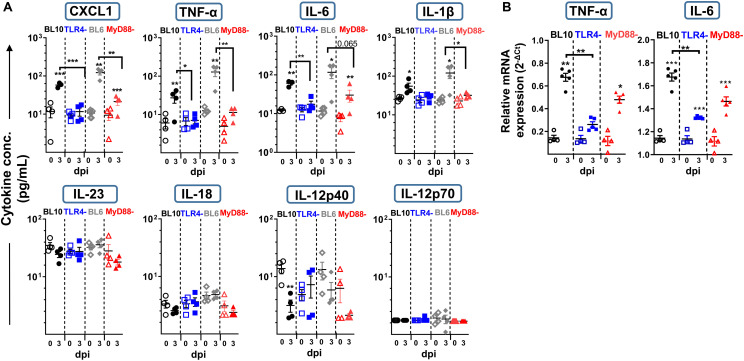
Lung cytokine production at 3 dpi in WT.BL10, TLR4^–/–^, WT.BL6 and MyD88^–/–^ *S. pneumoniae* (2 × 10^6^ CFUs) infected mice. Lung suspensions from control (empty symbols) and infected mice (filled symbols) were prepared and their supernatants analyzed. **(A)** CXCL1, IL-18, IL-23, IL-12p70, IL-6, TNF-α, IL-1β, and IL-12p40 protein levels were measured in LEGENDplex Cytokine Assays. **(B)** TNF-α and IL-6 gene expression in the lungs of WT.BL10, TLR4^–/–^, and MyD88^–/–^ measured by qPCR at 3 dpi using the Bio-Rad CFX Manager software to calculate the C_T_ for each reaction. The amount of each specific transcript in each cDNA sample was determined and expressed as the 2^–ΔCT^, relative to that of HPRT transcripts. The data are shown as in Figure 1B, representing 4-to-6 different samples evaluated in duplicate. The populations were compared with one-way ANOVA and with an unpaired two-tailed Student’s *t*-test: *p < 0.05, ** *p* < 0.01.

To further understand the susceptibility of the TLR4^–/–^ and MyD88^–/–^ mice to *S. pneumoniae* infection, we assessed the cytokines in the lung homogenates by 3 dpi (Figure 4A). There was a significant increase in pro-inflammatory cytokines like CXCL1, TNF-α, IL-6, and IL-1β in WT (BL10 and BL6) mice following *S. pneumoniae* infection, with CXCL1 being greater in infected WT.BL6 compared to WT.BL10 (*p* < 0.05), whereas no such increase in these cytokines was evident in TLR4^–/–^ infected mice. By contrast, there was a mild increase in CXCL1, TNF-α, and IL-6 in MyD88^–/–^ infected mice. The expression of TNF-α and IL-6 transcripts was assessed in lung homogenates from infected mice by qPCR at 3 dpi (Figure 4B), and while there was a strong increase in both cytokines in WT.BL10 and MyD88^–/–^ infected mice, this was not the case in TLR4^–/–^mice. An early increase in IL-23 and IL-12p40 has been described 24 h pi with *S. pneumoniae* (44, 45). There was no change in IL-23, IL-18 and IL-12p70 in the lungs of WT (BL10 and BL6), TLR4^–/–^ and MyD88^–/–^ mice at 3 dpi, although IL-12p40 diminished in the WT mice (Figure 4A). Thus, in the absence of TLR4 *S. pneumoniae* impaired the pro-inflammatory cytokine cascade in the lung (CXCL1, TNF-α, IL-6, and IL-1β) at 3 dpi. Furthermore, inflammatory cytokines were moderately affected in MyD88^–/–^ infected mice, indicating that the inflammatory response to *S. pneumoniae* is partially dependent on the MyD88 transduction machinery.

### *In vitro* NADPH Oxidase-Activity of Distinct Myeloid Populations After *S. pneumoniae* Challenge

**FIGURE 5:**
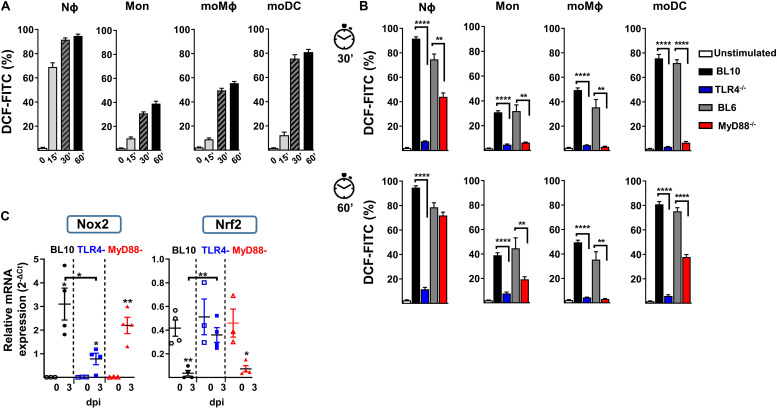
*In vitro* NADPH oxidase activity against heat-inactivated *S. pneumoniae* in lung cells. **(A)** Stained lung cells from WT.BL10 mice were labeled with the H_2_DCFDA-ROS sensitive probe (5 μM, DCF-FITC) and incubated with 2 × 10^6^ CFUs heat-inactivated *S. pneumoniae* (60 min, 60°C) for 0, 15, 30, and 60 min at 37°C before analyzing them by flow cytometry. **(B)** Detection of ROS in different myeloid populations from naïve mice using heat-inactivated *S. pneumoniae* at 30 and 60 min as in panel **(A)**. The bar graphs show the proportion of H_2_DCFDA^+^ cells. Unstimulated controls were shown for WT.BL10 animals. The data represents the mean ± SEM: WT.BL10 (*n* = 7), TLR4^–/–^ (*n* = 6), WT.BL6 (*n* = 5), and MyD88^–/–^ (*n* = 6). **(C)** Nox2 and Nrf2 gene expression by lung cells from WT.BL10, TLR4^–/–^, and MyD88^–/–^ mice measured by qPCR at 3 dpi, as described in Figure 4. The differences between the groups were analyzed by one-way ANOVA and with an unpaired two-tailed Student’s *t*-test: *p < 0.05, **p < 0.01, ***p < 0.001, ****p < 0.0001.

Nϕ and monocytes can activate NAPDH oxidase activity and generate ROS as part of the host’s defense against bacteria and parasites (46, 47). We studied the *in vitro* production of ROS in lung cell preparations after challenge with heat-killed *S. pneumoniae*, using the ROS-sensitive fluorescence probe H_2_DCFDA in Nϕ and in a variety of monocyte cell populations at different time points (Figure 5A). As expected, Nϕ rapidly produced large amounts of ROS in WT (BL10 and BL6) mice, with weaker induction and with slower kinetics in moDC, moMϕ and monocytes. The *in vitro* production of ROS in TLR4^–/–^ and MyD88^–/–^ mice differed significantly to that in WT (BL10 and BL6) mice (Figure 5B). In the case of TLR4^–/–^ lung cell preparations, ROS production by Nϕ and by the rest of monocyte populations was inhibited after 30 and 60 minutes. Furthermore, while the production of ROS was stronger in MyD88^–/–^ mice than in TLR4^–/–^ mice, in all cell types analyzed except in moMϕ, it remained weaker and with slower kinetics than that detected in WT.BL6 mice.

Regulation of the ROS-NAPDH pathway is mainly due to activation of the NADPH oxidase-2 (Nox2) transcription factor (48) and it is negatively regulated by Nrf2 (49). The transcripts of these two genes were quantified by qPCR in the three mouse models studied and Nox2 induction was evident in all infected mice, although it was weaker in the infected TLR4^–/–^ mice (Figure 5C). Furthermore, an important reduction of Nrf2 was evident in lung samples from WT.BL10 and MyD88^–/–^ infected mice but not in those from TLR4^–/–^ infected mice, in which it remained unchanged. Together, maintaining Nrf2 levels and weaker Nox2 induction may contribute to the diminished ROS production in TLR4^–/–^ infected mice.

## Discussion

The airway and alveolar epithelia represent the initial barrier for the recognition of respiratory pathogens to initiate a immune response against them. Bacterial airway infections, including those caused by *S. pneumoniae*, are a major cause of worldwide morbidity and mortality. Indeed, pneumonia is the leading cause of mortality in children below 5 years of age, although global child mortality has decreased substantially (50, 51). In addition, the mortality of individuals over 65 years due to pneumonia remains unchanged since 1990, in part because the coverage of pneumococcal vaccines remains low (52, 53). Nasopharyngeal colonization with *S. pneumoniae* in healthy children is common, indicating a role of the host response in avoiding invasive pneumococcal disease.

**FIGURE 6:**
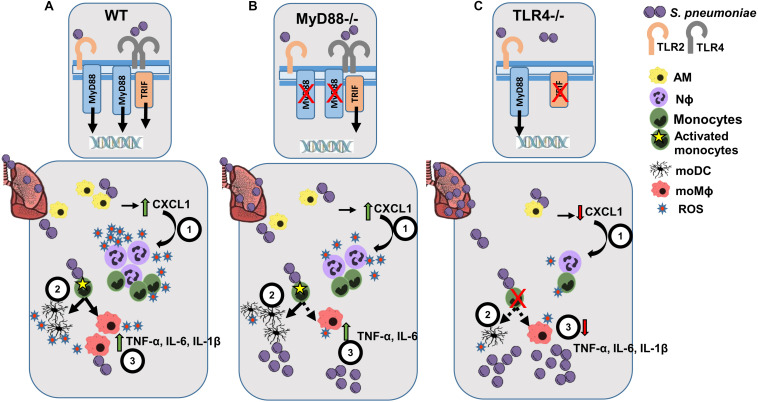
Scheme of the proposed lung IIR against *S. pneumoniae* in WT, MyD88^–/–^ and TLR4^–/–^ mice. **(A)** After infection of WT mice, TLR2 and TLR4 recognizes different PAMPs from *S. pneumoniae* activating the TRIF- and MyD88-dependent transduction pathways. After the initial bacterial encounter with AM cells, CXCL1 (1) is released that favors the early recruitment and activation of Nϕ cells, leading to substantial ROS production. (2) Differentiation of activated monocytes to moDCs and moMϕ helps to amplify the inflammatory response (3) through the secretion of a broad spectrum of cytokines and ROS production. **(B)** Infected MyD88^–/–^ mice can still signal through the TLR4-TRIF-dependent pathway, leading to a weak IIR response while maintaining an inflammatory cytokine environment. **(C)** TLR4^–/–^ infected mice experience impaired Nϕ recruitment and almost a complete blockade of monocyte differentiation, which leads to a very poor cytokine response and the development of a more intense bacterial burden.

Identifying mechanisms that can prevent bacterial lung infection is a global health priority, which requires a better understanding of the immune mechanisms involved in response to *S. pneumoniae* infection. The local IIR in the lung is important to combat *S. pneumoniae*, which involves the coordination of multiple cell populations and the activation of effector functions like phagocytosis, cytokine release, the complement cascade and antigen-presentation. Indeed, the IIR against *S. pneumoniae* is initiated through the recognition of PAMPs by TLRs, whose role and that of TLR-adaptor proteins and inflammasome complexes, has been studied using mouse models of infection (54–57). MyD88 and TLR4 are involved in local and systemic bacterial control, as well as in the recruitment of polymorphonuclear leukocytes in the lung (54). While TLR2 plays only a modest role in the immune response to *S. pneumoniae* in the respiratory tract, without affecting the overall antibacterial defense during infection (12), TLR9 has been implicated in the early clearance of bacteria, with no significant effect on local cytokine production (55). By contrast, TLR4, which signals both through MyD88-dependent and MyD88-independent (TRIF) pathways, also plays an important role by recognizing *S. pneumoniae* Ply and LTA (13) (Figure 6A). The impact of TLR recognition and signaling depends on the bacterial strain, and hence the pneumococcal serotype and the dose used to induce pneumonia may yield different results (54, 57). This study was carried out using a clinical serotype 3 encapsulated *S. pneumoniae* isolate, and by studying myeloid markers of activation and differentiation, we identified the myeloid events in the lung, local inflammatory responses and the ability to induce ROS production in response to infection of TLR4 and MyD88 deficient mice.

Consistent with these previous studies (54, 57), we show here that TLR4^–/–^ and MyD88^–/–^ infected mice have a higher bacterial load than their corresponding WT counterparts. Interestingly, lungs from infected MyD88^–/–^ mice had fewer bacterial CFUs than TLR4^–/–^ mice, which may indicate that the TRIF-dependent TLR4-signaling cascade plays an important role in bacterial clearance (Figures 6B,C). It was previously shown that the interaction between Ply and TLR4 during pneumococcal colonization of the nasopharynx is important for protection (14). Indeed, our results suggest an impaired immune response to *S. pneumoniae* in TLR4^–/–^ mice and to a lesser extent in MyD88^–/–^mice, compared to their corresponding WT genetic background (BL10 and BL6, respectively). Infected TLR4^–/–^ mice suffer a stronger reduction in AM cells and defective Nϕ induction. By contrast, the reduction of AM and the induction of Nϕ cells was similar in MyD88^–/–^ and WT.BL6 mice. As reported previously (20, 21), there was rapid differentiation of monocytes to moMϕ and moDC in *S. pneumoniae* infected WT animals. This induction of moMϕ was not observed in MyD88^–/–^ infected mice and it was severely dampened in *S. pneumoniae* infected TLR4^–/–^ mice. By contrast, moDC increased in MyD88^–/–^ infected mice to a similar extent as in the WT.BL6 mice, whereas there was no increase in moDC number in TLR4^–/–^ infected mice. The ratio of the cMO/ncMO populations in *S. pneumoniae*-infected TLR4^–/–^ mice reflected a cMO inflammatory monocyte profile, whereas that found in MyD88^–/–^ and in both the WT strains shifted toward a ncMO phenotype.

All these cellular defects after *S. pneumoniae* inoculation, mostly evident in TLR4^–/–^ mice, may contribute to the incomplete lung cytokine profile in these mice. Firstly, CXCL1 release, together with that of IL-1β and IL-6, plays a central role in Nϕ mobilization from the bone marrow, in Nϕ activation and in the control of bacterial dissemination in the lung after pneumococcal infection (58, 59). At 3 dpi, there was more CXCL1, TNF-α, IL6, and Il-1β in the lungs of WT (BL10 and BL6) mice, while IL-18 and IL-23 were not enhanced at this time, perhaps due to an earlier action of these cytokines at 24 h (44, 45, 48). Ply is not involved in the pneumococcal induction of IL-12p40 in murine Mϕ (60) and indeed, we detected less IL-12p40 in the lungs of infected WT (BL10 and BL6) mice relative to their uninfected WT controls. These changes may be indicative of a transient Th1 response occurring in the first 24 h pi, and related to the fast action of AM and NK cells as the first innate barrier to impede bacterial growth. In TLR4-defective mice there was no increase in CXCL1, TNF-α, IL6, and Il-1β at 3 dpi, while the increase in these cytokines was only mild in MyD88-deficient mice. Whether this transient inflammatory lung environment could influence the appearance of trained Mϕ cells (resident and bone marrow-derived), with an enhanced capacity to respond to a second PAMP-associated challenge, will be addressed in the near future. As such, an *in vivo* model of trained immunity should be useful in designing novel “trained-induced” based vaccines.

Bacterial phagocytosis and ROS production are essential to control *S. pneumoniae* pathogen-dissemination and overgrowth. Nϕ cells fulfill a central role in removing invading pneumococci through the rapid production of intracellular and/or extracellular ROS in response to PAMPs (61), or after phagocytosis (58). The release of Ply after autolysis of pneumococci activates NADPH oxidase and the intracellular production of ROS by Nϕ (33). Our results following *in vitro* stimulation with heat killed *S. pneumoniae* revealed that Nϕ were the fastest producers of ROS, although moMϕ and moDC can also produce notable amounts of ROS albeit with delayed kinetics and over longer periods. These cells not only play a key role in killing ingested pathogens but also in antigen presentation, cross-presentation and the activation of cytolytic T cells (62). Reactive oxygen species production by Nϕ and by all the monocyte-derived populations was severely inhibited in TLR4^–/–^ and to a lesser extent in MyD88^–/–^ mice, which may at least in part explain the rapid growth of *S. pneumoniae* in these animals. In humans, mitochondrial ROS production by AM cells was dampened in chronic obstructive pulmonary disease (COPD) patients but not in smokers, or in control individuals after *in vitro* challenge with *Haemophilus influenzae* or *S. pneumoniae* (63). Moreover, NADPH defects in humans favor fungal and bacterial infection (61). Likewise, the bactericidal activity of the fluoroquinolones levofloxacin and moxifloxacin against *S. pneumoniae* is related to the production of ROS by the bacteria, associated with transcriptional alterations induced by these fluoroquinolones (64–66). Moreover, oxygen-derived metabolites can also damage infected tissues, regulate immune functions and induce apoptosis (58, 67). The levels of the ROS inducer Nox2 and the antioxidant Nrf2 must be counterbalanced in order to overcome lung infection by *S. pneumoniae*, as demonstrated in WT and MyD88^–/–^ mice but not in TLR4^–/–^ mice. Indeed, targeting mitochondrial regulators of NADPH-oxidase production has been evaluated in leukemia cells (68), ischemic cerebral neurons (69), and human Nϕ cells after LPS activation (70). It is tempting to speculate that novel therapies of this type might benefit COPD patients, PRR-immunodeficient subjects, aged recurrent-pneumonia and patients with sepsis as promising alternatives to conventional antibiotic treatments. Likewise, such therapies could be useful to combat an important number of multi-resistant bacteria, with important implications for treatment and national healthcare budgets.

In summary, this study involved an in-depth analysis of different resident and recruited innate cell populations in the lung, addressing their cytokine production, their differentiation into distinct phenotypes and NADPH-oxidative metabolism, which in conjunction produces efficient pneumococcal clearance. All these host-mediated responses were severely impaired when TLR4 was absent, demonstrating the critical involvement of this receptor in the defense against the Gram-positive *S. pneumoniae*. In MyD88 deficient mice, a partial effect on the innate cell content, cytokine profile and ROS production was evident, indicating a role for either the TRIF-dependent and MyD88-independent signaling pathways, or alternatively, the implication of other PRRs like TLR9. As a result, our data highlight the importance of the TLR4-MyD88 axis in the recognition and efficient response to *S. pneumoniae* infection in the lung, raising new challenging questions that warrant further attention.

## Data Availability Statement

The datasets generated for this study are available on request to the corresponding authors.

## Ethics Statement

This study was carried out in accordance with EU and National Animal Care guidelines (directive 2010/63/EU and RD 53/2013). The procedures were approved by the Institutional Review Board at the ISCIII and the “Consejería de Medio Ambiente Comunidad de Madrid” (PROEX110/15 and PROEX021/18).

## Author Contributions

RS-T performed the experiments, contributed substantially to the analysis and interpretation of the data, and reviewed the manuscript. IC analyzed the data and reviewed the manuscript. JM, MR, and CR performed the experiments. MP and MA helped with the flow cytometry experiments. EC reviewed the manuscript. MF prepared the bacteria and performed the CFU analysis. AC provided bacteria and reviewed the manuscript. MG designed the experimental procedures, made substantial contributions to the analysis and interpretation of the data, and critically reviewed the manuscript. BA designed the experimental procedures, made substantial contributions to the analysis and interpretation of the data, and drafted and reviewed the manuscript. All authors contributed to the article and approved the submitted version.

## Conflict of Interest

The authors declare that the research was conducted in the absence of any commercial or financial relationships that could be construed as a potential conflict of interest.
