# Supplementary material for: The TLR4-MyD88 Signaling Axis Regulates Lung Monocyte Differentiation Pathways in Response to Streptococcus pneumoniae
Source: Front Immunol. 2020 Sep 16;11:2120. doi: 10.3389/fimmu.2020.02120 (PMC7525032; doi:10.3389/fimmu.2020.02120)
Supplement: Supplementary file 1 [file Image_1.pdf]

Supplementary Material

"The TLR4-MyD88 signalling axis regulates lung monocyte differentiation pathways in response to *Streptococcus pneumoniae*"

Rodrigo Sánchez-Tarjuelo, Isabel Cortegano, Juliana Manosalva, Mercedes Rodríguez, Carolina Ruíz, Mario Alía, María Carmen Prado, Eva M. Cano, María José Ferrándiz, Adela G. de la Campa, María Luisa Gaspar*, Belén de Andrés*.

*****Correspondence: Belén de Andrés or Maria Luisa Gaspar bdandres@isciii.es; mlgaspar@isciii.es.

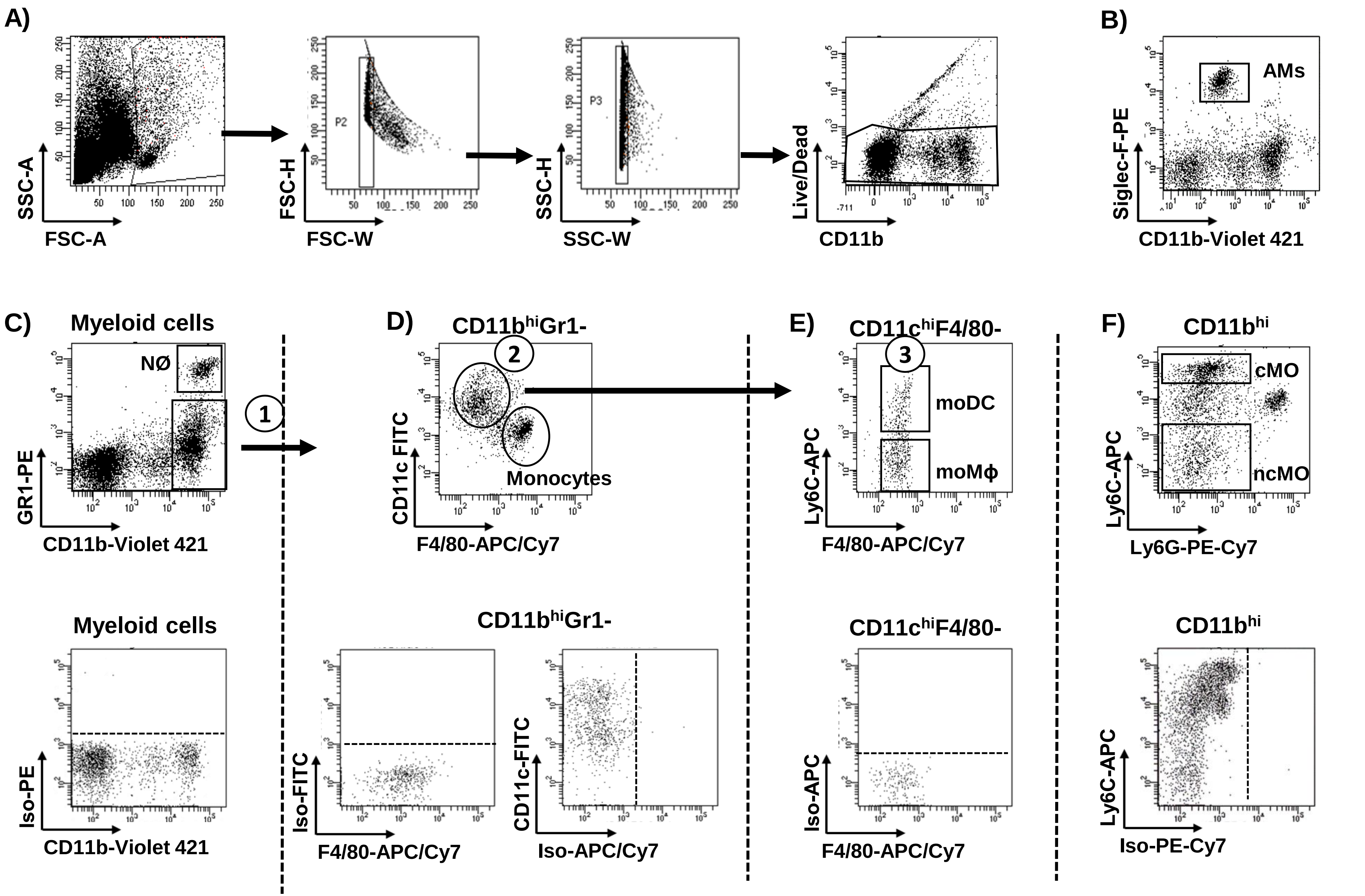
A)
SSC-A
FSC-A
FSC-H
FSC-W
P2
SSC-H
SSC-W
P3
Live/Dead
CD11b
B)
AMs
Siglec-F-PE
CD11b-Violet 421
C)
Myeloid cells
NØ
GR1-PE
CD11b-Violet 421
1
D)
CD11b^{hi}Gr1-
2
CD11c FITC
Monocytes
F4/80-APC/Cy7
E)
CD11c^{hi}F4/80-
3
moDC
moMϕ
Ly6C-APC
F4/80-APC/Cy7
F)
CD11b^{hi}
cMO
ncMO
Ly6C-APC
Ly6G-PE-Cy7
Myeloid cells
Iso-PE
CD11b-Violet 421
CD11b^{hi}Gr1-
Iso-FITC
F4/80-APC/Cy7
CD11c-FITC
Iso-APC/Cy7
CD11c^{hi}F4/80-
Iso-APC
F4/80-APC/Cy7
CD11b^{hi}
Ly6C-APC
Iso-PE-Cy7

Figure Supplementary 1. Lung suspensions were prepared and stained for flow cytometry analysis. Representative dot plots are shown in the figure, displaying at least 10^5 cells except in the Ly6C/F4/80 dot plot (10^3 cells). **A)** Myeloid-granulocyte populations were electronically gated on the basis of SSC-A and FSC-A . Doublets were discriminated using FSC-Height (H) versus FSC-Width (W) and SSC-H versus SSC-W strategy. Dead cells were discarded by staining with Fixable LIVE/ DEAD violet-510 kit, as a marker of fixed dead cells. **B**) Live cells (10^5-3 X 10^5 cells) were studied using different markers. Alveolar macrophages (AMs) were distinguished as SiglecF+CD11b-. C) A sequential gating strategy was used to identify myeloid CD11b+ populations expressing specific markers (upper dot plots) and gating boundaries of corresponding fluoresecence minus one (FMO) stainings (lower dot plots): 1.- Neutrophils (CD11b^{hi} GR1^{hi}) and monocytic cell populations (CD11b^{hi} GR1^{-}). D) 2.- Identification of the monocytic cell populations by means of F4/80 and CD11c expression. E) 3.- Monocytes can further differentiate into moMϕ by the differential expression of Ly6C (Gr1-CD11b^{hi}F4/80^{lo}CD11c+Ly6C-) and moDC (CD11b^{hi}F4/80^{lo}CD11c+Ly6C+). F) CD11b+ gated cells were analyzed for the classical monocytes (cMO) and non-classical monocytes (ncMO) phenotype characterization based on Ly6C and Ly6G expression: cMO (CD11b^{hi}Ly6C^{hi} Ly6G-) and ncMO (CD11b^{hi}Ly6C^{lo}Ly6G^{-}). Isotype fluorescence minus one (FMO) controls were displayed in the lower dot plots.

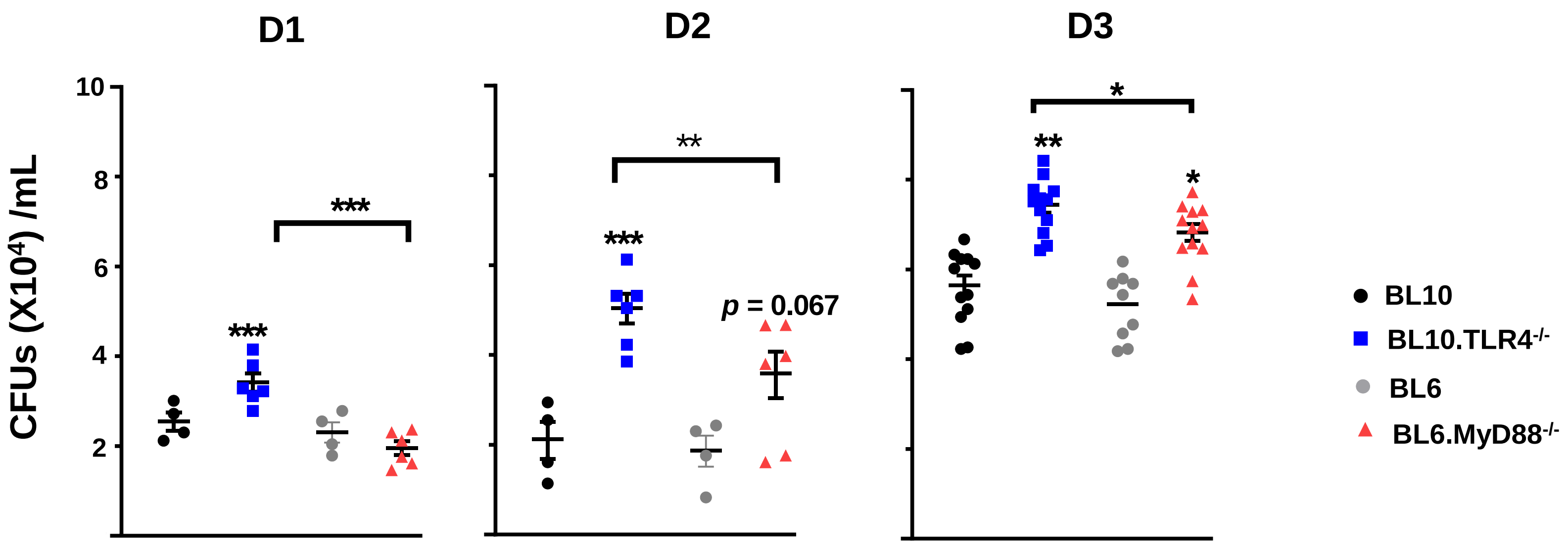

<u>Figure Supplementary 2</u> CFUs quantitation in lungs infected al 1, 2, and 3 dpi in WT.BL10 and TLR4-/- infected mice and in WT.BL6 and MyD88-/-. The data represent the individual measurements of three independent experiments. Shown inside are means ± SEM: day 1 and 2 dpi (n =6) each mice strain, 3 dpi (n =12) for all mice strains. The comparisons were made using an unpaired two tailed Student`s t-test: * p<0.05, ** p<0.01, *** p<0.001.

SUPPLEMENTARY MATERIALS AND METHODS

Extraction of single cell lung suspensions.

Extraction of adult mouse lungs (C57BL/6) was performed and organs were washed twice in PBS at 4 ºC.

Protocol A: One pulmonary lobe and cell suspensions were prepared by mechanical dissociation, disrupting the tissue and filtering through a 40 µm pore cell strainer (BD Biosciences). The filtrate was then centrifuged for 5 min at 110 g and 4 °C in order to obtain the lung cells.

Protocol B: The other lobe was cut into small pieces that were treated with collagenase D and DNase I in RPMI 1640 for 45 minutes at 37 °C on a shaker as described (*Wijburg et al.; Jungblut et al.*), disaggregating the tissue by pipetting. Suspensions were washed twice before filtering as described in Protocol A.

PROTOCOL	Absolute cell number/lobe (x 10^6)	%Myeloid cells (FSC-A/SSC-A)	%Live cells (IP-)	Abs. Num Myeloid live cells (x 10^3)	CD11b+ Abs. number (x 10^3)
A (n = 4)	81.7 ± 0,2	1.77 ± 0.9	72.2 ± 3	120 ± 15	114 ± 2.5
B (n = 4)	40.3 ± 0.32**	1.2 ± 0.3	68 ± 6	47 ± 25*	43 ± 13**

Comparisons were made using unpaired two-tailed Student`s t-test: *p <0.05, ** p<0.01,

There is an important diminution in the total number of cells and in the frequency of the myeloid cells (gated as described in Supplementary Figure 1) recovered using Protocol B in comparison with Protocol A. Both procedures had similar levels of live cells (gating out propidium iodide positive cells). Absolute numbers of myeloid cells and CD11b+ cells were diminished after Protocol B.

REFERENCES

-Wijburg OL, DiNatale S, Vadolas J, van Rooijen N, Strugnell RA. Alveolar macrophages regulate the induction of primary cytotoxic T-lymphocyte responses during influenza virus infection.*J Virol*. 1997;71(12):9450-9457.

-Jungblut M, Oeltze K, Zehnter I, Hasselmann D, Bosio A. Standardized preparation of single-cell suspensions from mouse lung tissue using the gentleMACS Dissociator. *J Vis Exp*. 2009;(29):1266. Published 2009 Jul 2. doi:10.3791/1266

-Shen C, Xu H, Alvarez X, Lackner AA, Veazey RS, Wang X. Reduced expression of CD27 by collagenase treatment: implications for interpreting b cell data in tissues. *PLoS One*. 2015;10(3):e0116667. Published 2015 Mar 10. doi:10.1371/journal.pone.0116667.

Supplementary Table 1. Antibodies used on flow cytometry.

ANTIBODY	CLONE	FLUOROCHROME	ORIGIN
CD11b	M1/70	VIOL421	BioLegend
CD11c	N418	FITC, PE/Cy7	eBioscience
CD87 (Ly6C)	HK1.4	APC	BioLegend
Ly6G	1A8	PE/Cy7	BioLegend
CD97 (GR1)	RB6-8C5	PE	BioLegend
F4/80	BM8	APC/Cy7	BioLegend
Siglec-F (CD170)	S17007L	PE	BioLegend
Isotype controls		FITC, PE, APC, PE/Cy7, APC/Cy7, VIOL421	BD
Fixable LIVE/ DEAD kit		VIOL510	ThermoFisher

FITC, fluorescein isotiocyanate; PE, R-phycoerythrin; APC, allophycocyanin; PE/Cy7, R-phycoerythrin conjugated with the tandem Cyanin 7. APC/Cy7, allophycocyanin conjugated with the tandem Cyanin 7.

Supplementay Table 2. List of primers used.

GENE	PRIMER 5´	PRIMER 3´	Temp	Refs
HPRT	GCCTGTATCCAACACTTCGA	TGTCATGAAGGAGATGGGAG	58º	1
TNF-α	GCCCAGACCCTCACACTCAG	AACACCCATTCCCTTCACAG	60º	2
IL-6	GAGGATACCACTCCCAACAGACC	AAGTGCATCATCGTTGTTCATACA	60º	3
Nrf2	GATCCGCCAGCTACTCCCAGGTTG	CAGGGCAAGCGACTCATGGTCATC	60º	4
Nox2	CAGGAACCTCACTTTCCATAAGAT	AACGTTGAAGAGATGTGCAATTGT	60º	5

1. de Andrés B, Prado C, Palacios B, Alía M, Jagtap S, Serrano N, Cortegano I, Marcos MA, Gaspar ML. Dynamics of the Splenic Innate-like CD19(+)CD45R(lo) Cell Population from Adult Mice in Homeostatic and Activated Conditions. *J Immunol*. 2012, 189:2300-8. doi: 10.4049/jimmunol.1200224.
2. Xu J, Eastman AJ, Flaczyk A, Neal LM, Zhao G, Carolan J, Malachowski AN, Stolberg VR, Yosri M, Chensue SW, Curtis JL, Osterholzer JJ, Olszewski MA. Disruption of Early Tumor Necrosis Factor Alpha Signaling Prevents Classical Activation of Dendritic Cells in Lung-Associated Lymph Nodes and Development of Protective Immunity against Cryptococcal Infection. MBio. 2016 Jul 12;7(4):e00510-16. doi: 10.1128/mBio.00510-16.
3. Anderson AE, Pratt AG, Sedhom MA, Doran JP, Routledge C, Hargreaves B, Brown PM, Lê Cao KA, Isaacs JD, Thomas R. IL-6-driven STAT signalling in circulating CD4+ lymphocytes is a marker for early anticitrullinated peptide antibody-negative rheumatoid arthritis. Ann Rheum Dis. 2016 Feb;75(2):466-73. doi: 10.1136/annrheumdis-2014-205850.
4. Ning Li, Jawed Alam, M. Indira Venkatesan, Arantza Eiguren-Fernandez, Debra Schmitz, Emma Di Stefano, Ndaisha Slaughter, Erin Killeen, Xiaorong Wang, Aaron Huang, Meiying Wang, Antonio H. Miguel, Arthur Cho, Constantinos Sioutas, Andre E. Nel *Nrf2* Is a Key Transcription Factor That Regulates Antioxidant Defense in Macrophages and Epithelial Cells: Protecting against the Proinflammatory and Oxidizing Effects of Diesel Exhaust Chemicals *J. Immunol.* 2004, 173 (5) 3467-3481. doi: 10.4049/jimmunol.173.5.3467.
5. Lv JZ, He XY, Wang HT, Wang ZH, Kelly GT, Wang XJ, Chen Y, Wang T, Qian ZQ. 2017. TLR4-NOX2 axis regulates the phagocytosis and killing of mycobacterium tuberculosis by macrophages. BMC Pulmonary Medicine, 17(1):194.
